# Biodegradable Films Targeting *Staphylococcus aureus*: Structure–Function Synergies and Interfacial Mechanisms

**DOI:** 10.3390/foods15040740

**Published:** 2026-02-17

**Authors:** He Dong, Yongli Wang, Wanru Zhao, Shiwei Yuan, Kai Song, Dongfang Shi

**Affiliations:** 1 School of Life Science, Changchun Normal University, Changchun 130032, China; mdonghe@163.com (H.D.); zhaowanrumei@163.com (W.Z.); ccsfysw00@163.com (S.Y.); 2 Department of Civil, Environmental, & Construction Engineering, Texas Tech University, Lubbock, TX 79409, USA; aenguswang8@gmail.com; 3 Institute of Innovation Science and Technology, Changchun Normal University, Changchun 130012, China

**Keywords:** biodegradable films, *Staphylococcus aureus*, antibacterial mechanisms, food packaging, biopolymer composites

## Abstract

*Staphylococcus aureus*, particularly its multidrug-resistant strains, poses a critical biological hazard throughout the global food supply chain, underscoring the need to transition from inert petroleum-based packaging to active, biodegradable alternatives. This review presents a comprehensive analysis of the structure function relationships and interfacial interaction mechanisms that govern polysaccharide-, protein-, and lipid-based films designed for the targeted inhibition of *S. aureus*. We critically evaluate the extent to which the intrinsic molecular features—such as the polycationic charge density of chitosan and the amphiphilic self-assembly of fatty acids—determine baseline antibacterial activity. A key contribution of this work is the elucidation of three synergistic pathways: physical barrier effects, chemical interference, and biological regulation. Furthermore, we discuss how composite systems, such as polysaccharide lipid hybrids and protein nanomaterial scaffolds, exploit charge complementarity and controlled-release kinetics to surpass the performance limitations of single-component materials. Finally, we address the critical trade-offs between mechanical integrity and antimicrobial efficacy, proposing a roadmap for intelligent, stimuli-responsive packaging that is capable of responding to microbial metabolic cues. Overall, this review provides a theoretical foundation for the rational design of high-performance biodegradable films to safeguard global food safety.

## 1. Introduction

*Staphylococcus aureus* (*S. aureus*) is a ubiquitous Gram-positive opportunistic pathogen and represents a critical biological hazard throughout the global food supply chain. In foodborne settings, *S. aureus* secretes heat-stable enterotoxins that can remain biologically active even after conventional thermal processing, making these toxins key etiological agents of foodborne intoxication [1]. Of particular concern is the global dissemination of methicillin-resistant *S. aureus* (MRSA) and other multidrug-resistant strains, which has substantially increased the complexity of clinical infection treatment and infection control (e.g., skin and upper respiratory tract infections) [2]. Global burden estimates indicate that foodborne diseases account for hundreds of millions of illnesses annually and impose a considerable socioeconomic burden on public health systems worldwide, underscoring the need to control major bacterial hazards such as *S. aureus* in the food supply chain, including toxin-mediated foodborne intoxications associated with *S. aureus* enterotoxin contamination [3]. In the foodborne context, *S. aureus* disease is primarily toxin-mediated intoxication caused by preformed enterotoxins rather than infection [1]. Even if viable cells are ingested, they do not normally establish infection, as most cells are inactivated in the gastrointestinal tract and cannot proliferate or spread in the host [1]. These alarming trends underscore the urgent need for effective and targeted antimicrobial strategies capable of suppressing *S. aureus* contamination throughout the food supply chain.

Beyond toxin production and antimicrobial resistance, the persistence of *S. aureus* in food environments is further exacerbated by its strong capacity for surface attachment and biofilm formation, which increases tolerance to environmental stresses and reduces the effectiveness of conventional sanitation and preservation measures. These traits collectively elevate the difficulty and cost of controlling *S. aureus* across processing, storage, and distribution, reinforcing the need for packaging materials that provide continuous, localized antimicrobial protection rather than relying solely on endpoint interventions.

In current food preservation systems, petroleum-based synthetic polymers such as polyethylene (PE) and polyvinyl chloride (PVC) remain dominant. These materials primarily function as passive physical barriers and generally lack intrinsic antimicrobial functionality. Moreover, their non-biodegradable nature contributes to persistent environmental pollution, commonly referred to as “white pollution,” which directly conflicts with global carbon neutrality goals and sustainable development strategies [4].

However, the transition to biodegradable active packaging still faces several practical “performance ceilings”. Many biopolymer matrices are intrinsically moisture-sensitive and may swell or lose barrier integrity under high-humidity food environments, which can trigger uncontrolled burst release of active agents and shorten the effective antimicrobial window. In addition, increasing antimicrobial loading often compromises mechanical strength, transparency, or processability, making it challenging to simultaneously achieve robust barrier performance, durability, and targeted antimicrobial efficacy under real-world supply-chain conditions. These coupled constraints motivate the development of a structure–mechanism–performance roadmap for rational design.

To address these dual challenges of food safety and environmental sustainability, the development of biodegradable packaging materials that combine environmental compatibility with active antimicrobial functionality has emerged as a major research frontier. Biodegradable films derived from natural macromolecules—including polysaccharides, proteins, and lipids—exhibit substantial potential owing to their inherent biocompatibility, renewability, and structural tunability [5]. Unlike inert synthetic polymers, these biopolymer matrices can form mechanically stable film networks through hydrogen bonding or electrostatic interactions while simultaneously serving as efficient carriers for natural antimicrobial agents such as plant essential oils, bacteriocins, and nanoparticles [6]. In certain cases, functional groups intrinsic to the biopolymers themselves (e.g., cationic moieties in chitosan) directly contribute to antimicrobial activity [7].

Distinct biopolymer matrices exhibit pronounced specificity in their antimicrobial pathways. For example, chitosan disrupts bacterial membrane integrity via its polycationic nature [7]; protein-based matrices such as gelatin can enable the encapsulation and controlled release of hydrophobic bioactive compounds within protein networks [8]; and chitosan-based active films impregnated with plant essential oils and phenolic extracts have been shown to suppress bacterial growth in ready-to-eat meat products during refrigerated storage [9]. These mechanistic differences highlight the importance of material selection and structural design in achieving targeted antimicrobial performance.

In this review, ‘biodegradable films’ refer to packaging-oriented films/coatings whose primary matrix is biodegradable under recognized disposal environments (e.g., industrial composting/soil), while ‘hybrid systems’ refer to biodegradable active layers applied on model or non-biodegradable substrates (e.g., PDMS/PU) for mechanistic proof-of-concept. Where non-biodegradable substrates are discussed, they are treated as platforms for interfacial/mechanistic studies rather than as biodegradable packaging matrices.

Although existing reviews have addressed biopolymer-based packaging materials or specific natural antimicrobial agents, few studies have systematically focused on *S. aureus* as a distinct target while providing a comparative analysis across polysaccharide-, protein-, lipid-based, and composite films. This review aims to bridge this gap by elucidating the interfacial interactions between diverse biopolymer films and *S. aureus*, encompassing both direct bactericidal effects and indirect regulatory mechanisms. Furthermore, this work systematically examines how material structure, processing strategies, and environmental factors collectively govern antimicrobial efficacy and durability. Ultimately, by revealing the structure–mechanism–performance relationships underlying biodegradable antimicrobial films, this review seeks to provide a theoretical foundation for the rational design of next-generation high-performance antimicrobial packaging materials.

## 2. Methodology

To ensure rigor, transparency, and comprehensive coverage, a structured literature search and selection process was conducted to minimize selection bias and evaluate the current landscape of biodegradable antimicrobial films targeting *Staphylococcus aureus*.

(1) Search strategy and database: A comprehensive search was performed using the Web of Science (Core Collection). Boolean operators were applied to combine thematic keywords as follows:

TS = (“biodegradable film*” OR “bio-based film*” OR “active packaging” OR “antimicrobial coating*”) AND TS = (“*Staphylococcus aureus*” OR “*S. aureus*”) AND TS = (“antimicrobial mechanism*”).

(2) Time window: Peer-reviewed research articles and review papers published from January 2000 to January 2026 were considered. This time window was selected to capture foundational concepts as well as recent advances in nanobiotechnology, active packaging, and interfacial antimicrobial mechanisms.

(3) Inclusion and exclusion criteria:

(a) Inclusion: (i) studies focusing on polysaccharide-, protein-, or lipid-based biodegradable matrices (including their composites); (ii) studies investigating structure–function relationships, interfacial interactions, or mechanistic pathways against *S. aureus*; and (iii) studies discussing synergistic enhancement in composite systems (e.g., charge complementarity, amphiphilic assembly, and controlled-release regulation).

(b) Exclusion: (i) studies exclusively based on petroleum-derived synthetic polymers (e.g., PE, PVC) without biodegradable matrices; and (ii) literature lacking clear mechanistic analysis and/or structural characterization relevant to film performance.

(4) Data synthesis: A thematic synthesis strategy was used to organize the selected literature. In total, over 110 relevant sources were selected and categorized into sections according to material type (polysaccharide-, protein-, and lipid-based films) and synergistic strategies (composite design and interfacial regulation) to elucidate the “structure–mechanism–performance” framework and its relevance to durable inhibition of *S. aureus* in food-packaging contexts.

In addition, reviewer-recommended studies and key references identified by backward citation screening were incorporated when they directly supported the targeted scope of this review.

## 3. Polysaccharide-Based Biodegradable Films

Polysaccharide-based polymers are cornerstone materials for the construction of functional food packaging films owing to their abundant natural availability, excellent biocompatibility, and tailorable biodegradability. Unlike synthetic polymers, polysaccharides possess densely distributed functional groups—such as hydroxyl, amino, and carboxyl moieties—that provide reactive sites for antimicrobial functionalization; some polysaccharides (e.g., chitosan) can also exhibit intrinsic antibacterial activity. These functional groups can act as molecular-level “active sites,” enabling direct interactions with microbial cell surfaces, while the three-dimensional polysaccharide network can simultaneously function as a microenvironmental modulator to facilitate the stabilization and controlled delivery of antimicrobial agents.

From a materials perspective, polysaccharide films offer dual functionality: on the one hand, they may exhibit contact-active antimicrobial effects through specific chemical functionalities (e.g., amino groups in chitosan); on the other hand, their interconnected polymer networks provide a versatile platform for modulating mass transfer, nutrient diffusion, and microbial adhesion. This section focuses on representative polysaccharide-based films and systematically analyzes their inhibitory activity against *S. aureus*, with emphasis on structure–function relationships and antibacterial mechanisms.

### 3.1. Chitosan Films: Multidimensional Antibacterial Systems Driven by Cationic Properties

**Figure 1:**
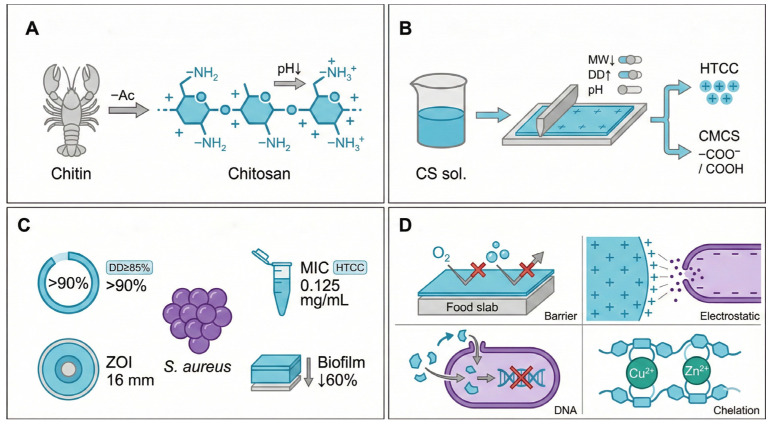
Molecular evolution, functionalization strategies, and multidimensional antibacterial mechanisms of chitosan films. (**A**) Origin and structural features; (**B**) Film formation and regulation; (**C**) Antibacterial performance metrics; (**D**) Synergistic antibacterial pathways.

Chitosan is currently one of the few naturally abundant alkaline polysaccharides and has been widely recognized as a benchmark material in active biodegradable packaging due to its excellent film-forming ability and broad-spectrum antibacterial activity. Structurally, chitosan is derived from chitin via partial deacetylation (Figure 1A). Although chitin is one of the most abundant biopolymers in nature, its high crystallinity, poor solubility, and low chemical reactivity severely restrict direct application [10,11,12]. In contrast, the deacetylation of chitin exposes primary amino groups at the C2 position of chitosan chains, which underpin its biological activity.

Under acidic conditions, these amino groups readily undergo protonation to form positively charged ammonium groups (–NH^3+^), imparting chitosan with pronounced polycationic characteristics [13]. This charge feature governs not only the solubility behavior of chitosan in aqueous systems but also its strong electrostatic affinity toward negatively charged bacterial cell surfaces, which are rich in teichoic acids and peptidoglycan. As a result, chitosan is one of the few film-forming biopolymers that exhibit intrinsic antibacterial activity without the need for additional antimicrobial agents [13].

The antibacterial performance of chitosan films is highly dependent on their molecular and physicochemical parameters, reflecting a pronounced structure–activity relationship (Figure 1B). Films are commonly fabricated via solution casting, and their efficacy is strongly influenced by molecular weight (MW), degree of deacetylation (DD), and environmental pH. Low-molecular-weight chitosan exhibits enhanced penetration capability, facilitating translocation across bacterial cell walls and intracellular interference [14,15]. Meanwhile, a higher DD corresponds to an increased density of amino groups and stronger positive charge, which generally enhances antibacterial potency [16].

To overcome the limited solubility of native chitosan under neutral conditions, chemical modification has been extensively explored. For instance, quaternization introduces permanent positive charges to yield quaternary ammonium chitosan (HTCC) [17], while carboxymethylation generates carboxymethyl chitosan (CMCS) with improved hydrophilicity (Figure 1B). These modifications not only enhance solubility and processability but also increase the density of active sites, thereby strengthening targeted antimicrobial performance [18].

Quantitative antibacterial assessments demonstrate that chitosan and its derivatives exhibit strong inhibitory effects against *S. aureus* (Figure 1C). Given the teichoic-acid- and peptidoglycan-rich surface of Gram-positive bacteria, the contact-active bactericidal action of chitosan is particularly pronounced [19]. When the DD is relatively high, chitosan typically shows strong inhibitory activity, and its antibacterial performance is closely associated with DD and MW [20,21]. Functionalized derivatives often outperform native chitosan; for example, quaternary ammonium chitosan derivatives (e.g., HTCC) have been reported to exhibit enhanced antibacterial activity and improved antibiofilm performance against *S. aureus* [17,19]. Recent work further suggests that tailoring chitosan network architecture in chitosan/PVA matrices can enhance antimicrobial performance and controlled delivery of active agents, providing additional design cues for chitosan-based antimicrobial films and coatings [22].

Morphological evidence further substantiates these findings. Zone-of-inhibition (ZOI) assays indicate that CMCS-based films can produce clear inhibition zones against *S. aureus* in vitro, supporting their contact-active antibacterial capability [23].

Mechanistically, the antibacterial activity of chitosan films arises from the synergistic contributions of four pathways: physical barrier formation, electrostatic disruption, intracellular interference, and metal ion chelation (Figure 1D) [14,15,19]. At the macroscopic level, chitosan films can form dense protective layers on food surfaces, restricting gas exchange (e.g., oxygen diffusion) and thereby modulating aerobic respiration [24]. In practical food preservation, combining chitosan-based treatments with modified atmosphere packaging has been applied to extend the shelf life of chilled chicken breast fillets [25]. Recent food-packaging studies have also demonstrated that chitosan-based composite films (e.g., levachitosan systems) can improve microbial quality and extend shelf life during refrigerated beef storage, further supporting their practical relevance for controlling *S. aureus*-related hazards [26]. At the microscopic scale, incorporating chitosan-based nanofibrous networks into packaging matrices can increase contact-active antimicrobial interfaces and inhibit foodborne pathogens, including *S. aureus* [27].

The dominant antibacterial mechanism involves electrostatic attraction between polycationic chitosan chains and negatively charged bacterial cell walls, leading to membrane destabilization and increased permeability, thereby promoting leakage of intracellular components [15,19]. Subsequently, chitosan-based nanocomposite films can further enhance antibacterial activity by intensifying contact-mediated membrane disruption and suppressing bacterial growth [28]. Related chitosan–inorganic nanocomposite matrices (e.g., chitosan—ZnS systems) have also been reported to enhance antibacterial and antibiofilm performance against resistant pathogens, offering complementary design insights for chitosan-based active films and coatings [29]. In addition, the amino and hydroxyl groups of chitosan exhibit strong chelation capacity toward essential metal ions (e.g., Cu^2+^, Zn^2+^), depriving bacteria of critical micronutrients and further disrupting enzymatic activity [30,31].

### 3.2. Cellulose-Based Films: From Structural Reorganization to Functional Carrier Design

**Figure 2:**
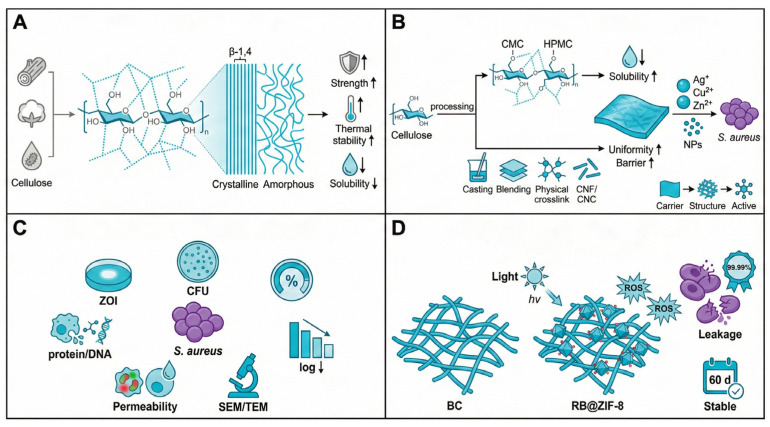
Hydrogen-bond network reconstruction, nanostructuring, and carrier-enabled antibacterial mechanisms of cellulose-based films. (**A**) Structural features and processing bottlenecks; (**B**) film formation and modification strategies; (**C**) antibacterial performance against *S. aureus*; (**D**) synergistic antibacterial pathways.

Cellulose is the most abundant linear polysaccharide in nature and is widely distributed in plant cell walls and bacterial extracellular products [32]. Structurally, cellulose consists of β-1,4-linked D-glucose units, with abundant hydroxyl groups along the polymer backbone that promote extensive intra- and intermolecular hydrogen bonding (Figure 2A) [33]. This highly ordered crystalline–amorphous multiphase architecture confers excellent mechanical strength and thermal stability but also renders cellulose insoluble and infusible in conventional solvents, thereby limiting direct film processing.

Consequently, the development of cellulose-based films typically begins with improving processability. A practical approach is to incorporate water-soluble cellulose derivatives such as carboxymethyl cellulose (CMC) as film-forming components or modifiers, which can improve film integrity and enhance barrier and mechanical performance in biopolymer-based film systems [34]. Alternatively, nanocellulose obtained through mechanical or chemical fibrillation (CNF/CNC) retains high crystallinity and a high aspect ratio, enabling the construction of dense “skeleton–filler” networks that simultaneously improve mechanical strength and barrier performance (Figure 2B).

Unlike chitosan, native cellulose generally exhibits limited intrinsic antibacterial activity and typically requires surface functionalization or incorporation of active agents to achieve effective antimicrobial performance [35]. Therefore, antibacterial strategies for cellulose-based films generally follow a functionalization paradigm, wherein cellulose serves as an inert yet highly effective carrier for antimicrobial agents, including metal ions (Ag^+^, Cu^2+^, Zn^2+^), essential oils, and antibacterial nanoparticles [36,37]. Within these systems, microstructural features of the cellulose matrix play a decisive role in regulating antibacterial efficacy.

Bacterial cellulose (BC) exemplifies this carrier-driven strategy. Its unique three-dimensional nanofibrillar network exhibits exceptional water-holding capacity and biocompatibility while providing a stable physical scaffold for immobilizing antimicrobial agents. For instance, in proof-of-concept antibacterial material designs, when BC is combined with composite nanofillers such as RB@ZIF-8, the synergistic effects of ZIF-8 porosity and BC network confinement enable bactericidal efficacy against *S. aureus* as high as 99.99%. Under photodynamic activation, severe cell wall rupture and intracellular leakage are observed, and antibacterial activity remains stable during prolonged storage (Figure 2C) [38].

From a mechanistic standpoint (Figure 2D), the antibacterial action of cellulose-based composite films arises from the integration of externally introduced active agents and nanoscale structural effects. Water-soluble derivatives such as CMC provide homogeneous film matrices suitable for dispersing polar antibacterial agents (e.g., nanosilver), facilitating contact-mediated release [39]. In contrast, nanofibrillated cellulose (NFC) enhances loading capacity through its high surface area and dense hydrogen-bonding network, forming a compact structure that limits mass transfer and permeation [40]. Through coordinated “carrier selection–structure construction–activity empowerment,” cellulose-based films compensate for their limited intrinsic activity by disrupting membrane permeability, promoting reactive oxygen species (ROS) generation, and disrupting cell wall integrity, ultimately achieving effective inhibition of *S. aureus*.

From an application perspective, different cellulose forms play distinct functional roles in antibacterial film systems. Water-soluble cellulose derivatives (e.g., carboxymethyl cellulose, CMC) are well suited as dispersion and delivery matrices for antibacterial agents. Their ability to form uniform films is facilitates contact-mediated release of active components; however, further improvements in water resistance and mechanical performance remain necessary. Bacterial cellulose (BC), owing to its high purity and three-dimensional nanofibrillar network, exhibits excellent biocompatibility and physical confinement capability, making it an ideal carrier for constructing high-efficiency antibacterial composite systems, although the cost of large-scale production remains relatively high. In contrast, nanofibrillated cellulose (NFC) forms a dense hydrogen-bonded networks via high aspect-ratio fibrils, which not only provides a degree of physical barrier by limiting mass transfer and permeation but also markedly enhances the mechanical and barrier properties of films mechanical strength and barrier performance; nevertheless, colloidal stability remains a key challenge for practical deployment. A comparative summary of these cellulose materials in terms of structural characteristics, antibacterial modes of action, and application-specific advantages and limitations is provided in Table 1.

## 4. Protein-Based Biodegradable Films

Protein-based materials, including collagen, whey protein, and soy protein, have been extensively explored for the fabrication of functional antimicrobial films due to their favorable film-forming ability, biodegradability, and biocompatibility [41]. In contrast to polysaccharide-based systems, the antibacterial performance of protein-based films is largely governed by their structural tunability and controlled loading/release capability for antimicrobial agents.

At the molecular level, proteins are composed of amino acids linked via peptide bonds and contain a diverse array of functional groups, such as amino, carboxyl, hydroxyl, and thiol moieties [42]. These groups enable proteins to form well-defined three-dimensional conformations through hydrogen bonding and hydrophobic interactions, with disulfide bond formation further contributing to folding stability [43]. Such structural versatility not only underpins robust film formation but also allows protein matrices to interact with bacterial surfaces and to encapsulate, stabilize, and control the release of antimicrobial agents, thereby exerting inhibitory effects against *S. aureus*.

### 4.1. Collagen-Based Films

**Figure 3:**
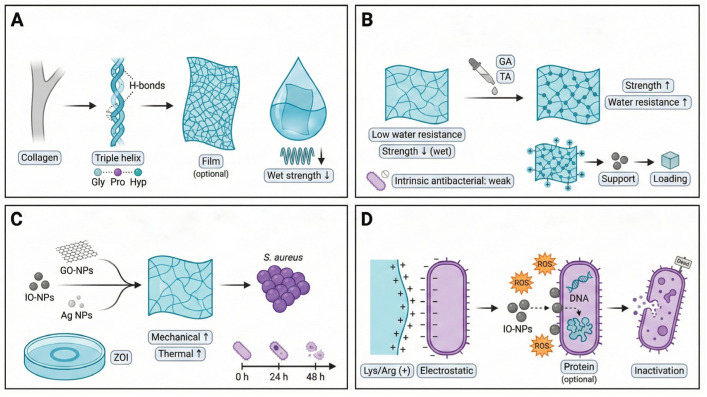
Structural basis, reinforcement strategies, and nanocomposite antibacterial mechanisms of collagen films. (**A**) Collagen molecular structure; (**B**) Film reinforcement via crosslinking and carrier effects; (**C**) Antibacterial performance of nanocomposites; (**D**) Synergistic mechanisms involving electrostatics and ROS generation.

Collagen is the most abundant structural protein in animal connective tissues [44]. It has also been widely explored for biodegradable and biocompatible film-based materials due to its favorable film-forming capacity [45]. Structurally, collagen molecules consist of three polypeptide chains assembled into a stable right-handed triple-helix, with sequences rich in glycine, proline, and hydroxyproline residues [46,47]. This unique molecular architecture imparts collagen with intrinsic structural stability (Figure 3A) [47].

Although collagen molecules can assemble into continuous film networks, pure collagen films exhibit pronounced mechanical weakening under humid conditions, which restricts their direct application [48]. Early studies initially focused on verifying film-forming feasibility and biocompatibility, while subsequent efforts addressed mechanical and water-resistance limitations through chemical crosslinking strategies [49]. Crosslinkers such as glutaraldehyde increase network density and stability, thereby enhancing tensile strength and moisture resistance (Figure 3B) [50]. However, for direct food-contact packaging, the use of glutaraldehyde is constrained by migration and toxicological concerns; therefore, food-grade crosslinkers (e.g., tannic acid, organic acids, or genipin) are preferred for packaging-oriented formulations.

However, native collagen films possess limited intrinsic antibacterial activity, which constrained their early use in food packaging and biomedical protection. With growing interest in multifunctional materials in the early 2000s, research attention shifted toward exploiting collagen as a carrier platform. Positively charged amino acid residues (e.g., lysine and arginine) within collagen chains may contribute to electrostatic interactions with negatively charged bacterial membrane components, providing a chemical basis for integrating collagen with exogenous antimicrobial agents. In practice, antibacterial performance is often achieved by incorporating functional nanofillers (e.g., graphene oxide and iron oxide nanoparticles) into collagen films [51].

Over the past decade, advances in nanotechnology have driven collagen films from passive carriers toward active antimicrobial systems [52]. Incorporation of functional nanomaterials—such as graphene oxide (GO-NPs), iron oxide nanoparticles (IO-NPs), and silver nanoparticles—has enabled simultaneous reinforcement of mechanical and thermal properties while imparting strong antibacterial activity (Figure 3C). Time-dependent antibacterial evaluations indicate that iron-oxide nanoparticle functionalized biopolymer matrices, including collagen-based composites, can enhance inhibitory activity against *S. aureus* over time, as reflected by viability-based assays and inhibition-zone measurements [53,54,55].

Mechanistically (Figure 3D), the enhanced antibacterial performance of collagen-based nanocomposites arises from the synergy between electrostatic interactions and ROS-mediated oxidative damage associated with iron oxide nanoparticles [56]. Positively charged residues in collagen weaken bacterial membranes via electrostatic attraction, while embedded nanoparticles generate reactive oxygen species (ROS) that induce oxidative damage to bacterial DNA, proteins, and membrane lipids. IO-NPs, in particular, are believed to internalize into bacterial cells and, together with ROS generation, disrupt cell walls and DNA, ultimately leading to cell inactivation or death [57].

### 4.2. Whey Protein-Based Films

Whey protein, a major byproduct of cheese production, is rich in functional components such as β-lactoglobulin (β-LG) and α-lactalbumin (α-LA). Its molecular structure contains diverse amino acid residues, free thiol groups, and hydrophobic domains, which collectively provide the physicochemical basis for film formation and functionalization [58].

**Figure 4:**
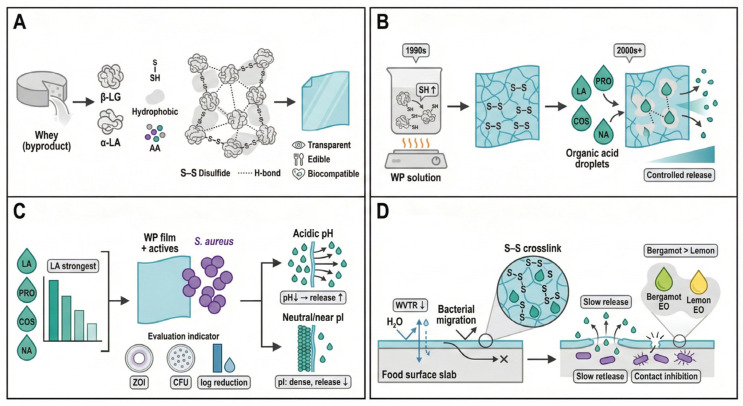
Thermal gelation, encapsulation behavior, and environment-responsive release mechanisms of whey protein films. (**A**) Heat-induced assembly; (**B**) film formation and functionalization strategies; (**C**) antibacterial performance and influencing factors; (**D**) integrated antibacterial mechanisms.

Early studies primarily employed heat-induced denaturation to fabricate whey protein films. Thermal treatment unfolds protein conformations, exposing reactive groups that subsequently form intermolecular disulfide bonds, hydrogen bonds, and hydrophobic interactions. These interactions generate stable three-dimensional networks with good transparency, mechanical integrity, and food-contact suitability (Figure 4A) [59,60].

With the evolution of functional food packaging concepts, research in the 21st century increasingly emphasized composite design. Whey protein matrices were utilized to encapsulate antimicrobial and antioxidant agents, leveraging reactive thiol groups and hydrophobic regions to bind organic acids (e.g., lactic acid) and other bioactive molecules. Such interactions enable effective stabilization and controlled release of antimicrobial compounds [61].

In antibacterial applications, whey protein films are commonly employed as carriers for organic acids, natural antimicrobials, or bioactive agents to enhance inhibition of Gram-positive bacteria. Studies targeting *S. aureus* demonstrate that films incorporating lactic acid (LA), propionic acid (PRO), chitosan oligosaccharides (COS), or natamycin (NA) exhibit varying degrees of antibacterial activity. Among these, lactic-acid-loaded whey protein films produce relatively large inhibition zones, suggesting sustained release facilitated by protein–acid interactions [61].

Notably, the release and antimicrobial performance of whey protein films are influenced by the physicochemical state of the film matrix (Figure 4C). Incorporation of organic acids and other antimicrobials can alter moisture content, solubility, and water vapor permeability, which in turn affects migration/release behavior and antibacterial efficacy [61].

Overall antibacterial performance results from the coupling of physical barrier effects and controlled release mechanisms (Figure 4D). For example, whey protein concentrate edible films incorporated with liquid smoke have been shown to exhibit antimicrobial activity in agar diffusion tests while maintaining (or even improving) mechanical properties, highlighting the importance of protein–additive interactions in balancing functionality and durability [62].

When essential oils are incorporated, hydrophobic protein domains stabilize volatile compounds and enable sustained release. Comparative studies indicate that bergamot essential oil exhibits stronger antibacterial activity than lemon oil when encapsulated within whey protein matrices, highlighting the importance of compatibility between active agents and protein microenvironments [63].

### 4.3. Soy Protein-Based Films

**Figure 5:**
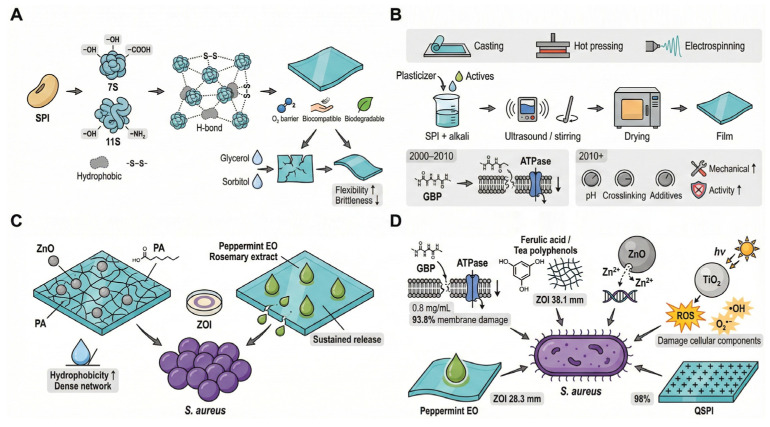
Globulin dissociation, process evolution, and endogenous–exogenous synergistic antibacterial systems of soy protein films. (**A**) Composition and film-forming basis; (**B**) evolution of film-forming processes; (**C**) representative antibacterial systems and their efficacy; (**D**) multitarget antibacterial mechanisms.

Soy protein is a widely available, low-cost, and renewable plant-derived polymer, most commonly utilized in the form of soy protein isolate (SPI). SPI primarily consists of 7S and 11S globulins and contains abundant hydroxyl, carboxyl, and amino groups, along with hydrophobic domains and free thiol groups. These features enable SPI to form continuous three-dimensional networks via hydrogen bonding, hydrophobic interactions, and disulfide crosslinking in the presence of plasticizers such as glycerol or sorbitol (Figure 5A).

In most SPI film studies, including graphene-modified systems, solution casting is commonly used: SPI is typically dissolved under mildly alkaline conditions, followed by dispersion/homogenization and drying to form films [64]. As summarized in (Figure 5B), early work focused on identifying naturally antibacterial components in the SPI system; for example, glycinin basic peptide (Glycinin Basic Peptide, GBP) has been reported to have the potential to disrupt cell membranes or inhibit intracellular enzyme activity [65]. More recently, the research paradigm gradually shifted toward integrated “formulation–process–function” optimization, aiming to simultaneously address the bottlenecks of relatively weak mechanical strength and poor water resistance in plant-protein films by adjusting solution pH and introducing crosslinkers or functional fillers, thereby achieving dual improvements in structural stability and active functionality [66].

The inhibitory effect of SPI-based film systems on *S. aureus* is not driven by a single mechanism; rather, it results from the combined contributions of multiple pathways, including “endogenous active fragments,” “composite synergistic effects,” “construction of charged interfaces,” and “sustained-release delivery” (Figure 5C,D). First, endogenous antimicrobial peptides present in the SPI system provide baseline antibacterial activity. In vitro studies have shown that GBP at 0.8 mg/mL can damage the cell membranes of 93.8% of *S. aureus* cells and trigger intracellular K^+^ leakage and ATPase inactivation, directly disrupting bacterial energy metabolism.

Second, to overcome the performance limits of single protein films, chemical crosslinking and functional enhancement using plant polyphenols (e.g., ferulic acid and tea polyphenols) have become a mainstream strategy. Phenolic compounds can interact with protein side chains via hydrogen bonding and hydrophobic interactions and promote crosslinking, forming a more stable network and improving the interfacial distribution of active components. For instance, SPI/CNF/ferulic acid (FA) composite films have been reported to exhibit improved packaging properties and enhanced antibacterial efficacy, consistent with FA-mediated crosslinking that densifies the protein network [67].

Third, the incorporation of inorganic nanomaterials markedly enhances bactericidal strength. Nano-ZnO can release Zn^2+^ and penetrate the bacterial cell wall, react with cytoplasmic components, and inhibit DNA replication [68]; meanwhile, TiO_2_ under photocatalytic conditions generates reactive oxygen species (ROS, such as •OH and O^2−^), inducing oxidative damage to proteins and lipids and ultimately leading to bacterial cell death. The catalytic activity of TiO_2_ can be further optimized through elemental doping: For example, vanadium-doped TiO_2_ has been applied as an electrocatalyst for azo-dye degradation, and characterization and performance results indicate enhanced catalytic degradation efficiency, providing indirect evidence for improving the activity of TiO_2_-based nano-additives in soy protein films [69]. This result is presented as evidence of enhanced catalytic/ROS-generating potential and does not substitute for direct antimicrobial evidence under film-relevant conditions. In addition, pronounced synergistic enhancement can occur between natural extracts and nanoparticles; for example, a composite system of mangosteen peel extract and ZnO can amplify antibacterial effects through multiple mechanisms [70].

Finally, for long-acting antibacterial needs, chemical modification and sustained-release strategies offer effective solutions. Quaternized soy protein isolate (QSPI) prepared via quaternization modification bears stable positive charges on its surface, enabling efficient capture and disruption of bacteria through electrostatic adsorption, and can achieve an inhibition rate greater than 90% against *S. aureus* [71]. At the same time, encapsulation within the protein network can effectively delay the volatilization and migration of hydrophobic essential oils. For example, peppermint essential oil itself exhibits potent antibacterial and anti-biofilm activities against *S. aureus* by damaging membrane integrity and promoting leakage of intracellular constituents (e.g., nucleic acids, proteins, and ATP), supporting its use as a promising hydrophobic active agent for incorporation into SPI-based films [72].

A comprehensive comparison of the three representative protein-based films discussed above reveals that their inhibitory effects against *S. aureus* differ markedly in terms of material origin, mechanisms of action, and application scenarios. Collagen-based films often serve as functional carrier platforms, achieving synergistic antibacterial activity through the loading of nanomaterials that induce reactive oxygen species (ROS) generation. Whey protein films, by contrast, rely on heat-induced and disulfide-crosslinked network structures and are particularly effective in encapsulating organic acids or essential-oil-based antibacterial agents and enabling their controlled release. Soy protein films not only possess intrinsic antibacterial activity derived from endogenous antimicrobial can also further enhance antibacterial potency and durability through chemical modification or incorporation of inorganic nanomaterials. A systematic comparative summary of the antibacterial pathways, structural advantages, and application potential of these three film types is presented in Table 2.

## 5. Antibacterial Properties of Lipid-Based Biodegradable Films

Lipid-based materials, including fatty acids, waxes, and glycerides, occupy a unique position within biodegradable film systems owing to their pronounced hydrophobicity. In most film formulations, lipid components form discontinuous to semi-continuous hydrophobic phases that effectively impede moisture migration and microbial penetration. In addition to this physical barrier function, specific lipid molecules—particularly medium-chain and unsaturated fatty acids—exhibit intrinsic antibacterial activity.

The long hydrocarbon chains and functional head groups (e.g., carboxyl and ester moieties) of lipid molecules enable strong interactions with the cytoplasmic membrane of *S. aureus*. Consequently, lipid-based films can modulate the local microenvironment through structural densification while simultaneously inducing membrane dysfunction through direct lipid–lipid interactions, thereby achieving effective antibacterial performance.

### 5.1. Fatty Acid-Based Films: Interfacial Assembly and Membrane-Targeted Interference

**Figure 6:**
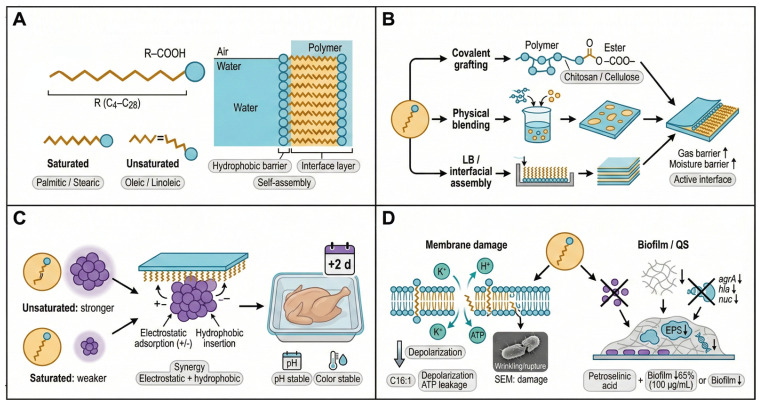
Amphiphilic assembly, membrane insertion, and quorum sensing interference mechanisms of fatty acid-based films. (**A**) Molecular structure and interfacial behavior; (**B**) film construction and immobilization; (**C**) antibacterial action of unsaturated fatty acids; (**D**) dual antibacterial mechanisms.

Fatty acid-based films are constructed using fatty acids or their derivatives as core functional units. Fatty acids are long-chain carboxylic acids that can be classified as saturated (e.g., palmitic acid and stearic acid) or unsaturated (e.g., oleic acid and linoleic acid) based on the degree of unsaturation. Their amphiphilic molecular architecture, consisting of hydrophilic head groups and hydrophobic tails, promotes spontaneous orientation and aggregation at interfaces (Figure 6A).

Exploiting this self-assembly behavior, fatty acids can be organized into ordered monolayer or multilayer structures using Langmuir–Blodgett (LB) techniques or solution-casting-induced interfacial alignment. Such structural organization enhances gas barrier and moisture resistance while facilitating interactions between hydrophobic tails and bacterial membranes. In addition to physical blending, fatty acids can be covalently immobilized onto polysaccharide backbones (e.g., chitosan or cellulose) via esterification or acylation, yielding polymer films with active functional interfaces (Figure 6B).

Antibacterial efficacy is strongly influenced by fatty acid molecular conformation (Figure 6C). Unsaturated fatty acids generally exhibit greater inhibitory activity against Gram-positive bacteria due to the presence of double bonds, which increase molecular flexibility and facilitate insertion into bacterial lipid bilayers. In contrast, long-chain saturated fatty acids display weaker activity but can still perturb transmembrane transport through hydrophobic interactions.

Notably, antibacterial fatty acids show enhanced efficacy against *S. aureus* biofilms, destabilizing hydrophobic multicellular aggregates and dispersing established biofilms, which contributes to higher inhibition and prolonged antibiofilm activity [73]. From an application perspective, fatty-acid-based interventions have demonstrated commercial potential; for example, lauric acid in a cooked chicken food model system prolonged shelf life by about two days at 4 °C without adverse effects on pH or color stability [74].

Mechanistically, fatty acid-based films primarily exert antibacterial effects through membrane disruption, accompanied by additional regulation of bacterial signaling pathways (Figure 6D). Hydrophobic tails insert into phospholipid bilayers, disturbing lipid packing and generating localized defects. This process increases membrane permeability, induces leakage of ions (e.g., K^+^, H^+^) and ATP, and disrupts electrochemical gradients and energy metabolism. Experimental evidence shows that palmitoleic acid (C16:1) rapidly induces membrane depolarization and triggers leakage of intracellular solutes including ATP; electron microscopy supports membrane permeabilization without catastrophic morphological collapse [75].

Beyond physical damage, fatty acids can interfere with quorum sensing (QS) systems, thereby inhibiting bacterial adhesion and biofilm formation. Unsaturated fatty acids alter surface hydrophobicity or block signaling molecule transmission, preventing initial attachment and extracellular polymeric substance (EPS) secretion. Myristoleic acid inhibits biofilm formation at concentrations as low as 1 µg/mL [76]. In-depth studies targeting *S. aureus* have demonstrated that petroselinic acid can downregulate the transcription of the quorum-sensing regulatory gene agrA as well as virulence-associated genes (such as hla and nuc), thereby reducing the secretion of α-hemolysin and lipase [77]. More generally, bacteria can undergo broad transcriptomic reprogramming as an adaptive strategy (illustrated in *Acinetobacter baumannii* during growth-phase transitions), but this example is provided only as background and not as interspecies evidence for *S. aureus* QS regulation [78]. Together, these findings support a transcription-linked mechanism in which fatty acids suppress virulence by interfering with the expression of key regulatory genes in *S. aureus* [77].

In addition, fatty-acid-based films may inhibit bacterial adhesion and aggregation by disrupting the stability of the extracellular polymeric substance (EPS) matrix within biofilms. Previous studies have reported that petroselinic acid can inhibit more than 65% of biofilm formation at a concentration of 100 µg/mL and remains effective against methicillin-resistant *S. aureus* (MRSA) strains [77].

### 5.2. Wax-Based Films: Construction of Hydrophobic Barriers and Anti-Adhesive Interfaces

**Figure 7:**
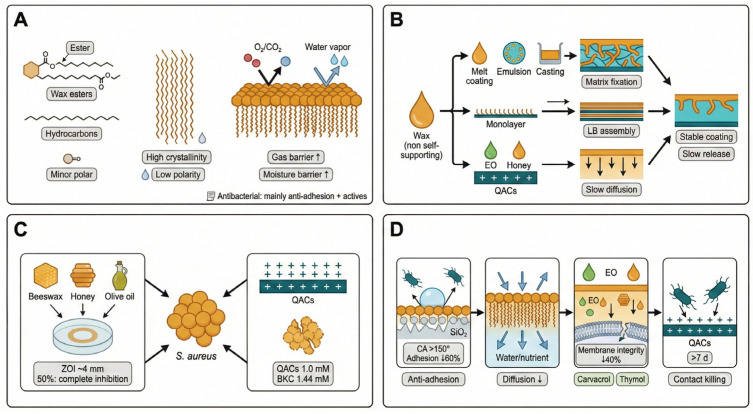
Composition, composite strategies, and anti-adhesive/antibacterial mechanisms of wax-based films. (**A**) Crystalline barrier formation; (**B**) biomimetic surface construction; (**C**) antibacterial performance; (**D**) synergistic antibacterial pathways.

Wax-based films are formulated using natural waxes or wax-derived components as the primary hydrophobic phase. Chemically, waxes consist predominantly of long-chain lipids, including wax esters formed from fatty acids and fatty alcohols, hydrocarbons, and minor polar constituents. Their high crystallinity and low polarity promote dense packing and orientation at interfaces, leading to compact hydrophobic layers (Figure 7A).

Although waxes significantly enhance gas and moisture barrier properties, their poor self-supporting film-forming ability necessitates composite strategies. Recent quantitative mass-transfer measurements further show that candelilla-wax/hydrocolloid edible films can markedly reduce VOC permeability while improving water vapor control, providing direct evidence for wax-based barrier enhancement in food packaging [79]. Typically, waxes are incorporated into polysaccharide or protein matrices via melt coating, emulsion dispersion, or interfacial assembly. Alternatively, Langmuir–Blodgett techniques enable controlled formation of ordered mono- or multilayers (Figure 7B). To move beyond passive barrier roles, wax matrices are often functionalized with natural antimicrobials (e.g., essential oils or honey) or cationic agents, exploiting diffusion resistance and hydrophobic encapsulation for interfacial enrichment and sustained release.

Antibacterial activity against *S. aureus* generally arises from the combined effects of structural barriers and active agent release (Figure 7C). For example, a 1:1 mixture of beeswax, honey, and olive oil produces inhibition zones of approximately 4 mm and achieves complete growth suppression at 50% concentration [80]. Incorporation of quaternary ammonium compounds (QACs) further enhances efficacy; QAC-containing biobased wax coatings completely inactivate *S. aureus* at 1.0 mM, outperforming the commercial benchmark quaternary ammonium compound benzalkonium chloride (BAC) under the same test conditions (1.44 mM) [81].

At the mechanistic level (Figure 7D), wax-based films operate through anti-adhesive physical barriers and chemical interference. Hydrophobic surfaces significantly reduce initial bacterial adhesion, while microstructural control yields superhydrophobic interfaces. Beeswax–SiO_2_ composite coatings achieve contact angles exceeding 150° and significantly reduce bacterial adhesion [82]. Beeswax has been reported to exhibit antimicrobial effectiveness, and these inhibitory effects can be enhanced synergistically when combined with other natural products such as honey or olive oil [83].

When combined with active agents, sustained release becomes the dominant factor for long-term antibacterial efficacy. For instance, carnauba-wax/chitosan coatings incorporating oregano essential oil have been shown to reduce microbial decay in fresh produce, supporting the use of wax–biopolymer matrices as carriers for hydrophobic antimicrobials in food preservation [84]. QAC-modified wax coatings destabilize membrane potential via electrostatic interactions, ultimately inducing cell lysis. Importantly, wax matrices can prolong the release of hydrophobic active agents, enabling antibacterial activity to persist over extended periods.

### 5.3. Glyceride-Based Films: Amphiphilicity-Driven Membrane Targeting and Signal Interference

Glyceride-based materials are esters formed between glycerol backbones and fatty acid chains. Depending on the degree of esterification, they are classified as mono-, di-, or triglycerides. Monoglycerides exhibit the most pronounced amphiphilicity due to the coexistence of hydrophilic glycerol head groups and hydrophobic fatty acid tails, enabling spontaneous self-assembly at aqueous–organic interfaces.

**Figure 8:**
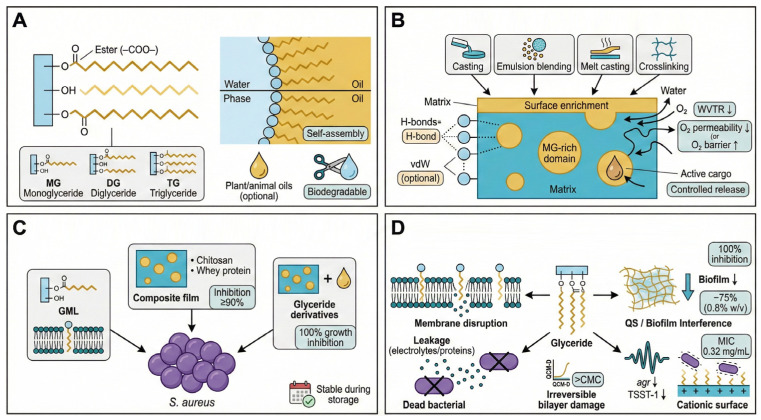
Self-assembly behavior, microphase structure, and membrane-solubilization mechanisms of glyceride-based films. (**A**) Amphiphilic structure and interfacial behavior; (**B**) film microstructure; (**C**) potent antibacterial activity; (**D**) dual mechanisms: membrane disruption and QS/biofilm interference.

In film fabrication (Figure 8A,B), glycerides derived from natural sources such as coconut, palm, and castor oils are incorporated using solution casting, emulsion blending, or melt processing. Within composite systems, glycerides play dual roles: hydrophobic tails enhance moisture and oxygen barrier properties, while residual hydroxyl groups form hydrogen-bond networks with polysaccharide or protein matrices, improving flexibility, interfacial stability, and controlled release capacity.

Monoglycerides, particularly glycerol monolaurate (GML), exhibit strong antibacterial activity against *S. aureus* (Figure 8C). MIC- and growth-inhibition-based evaluations show that monoglycerides (including GML) can completely inhibit *S. aureus* growth at sufficiently high concentrations [85,86]. Beyond bactericidal effects, glycerides also suppress biofilm formation. For example, sophorolipids have been reported to inhibit microbial biofilms on medical-grade silicone surfaces, indicating that amphiphilic lipid-based agents can suppress biofilm formation [87].

Structural modification further enhances activity. Introduction of cationic groups into glyceride-containing systems strengthens electrostatic interactions. Castor-oil-derived (glyceride-based) moisture-cured reactive polyurethanes provide a film-forming matrix that can be further functionalized for antimicrobial applications [88].

Antibacterial mechanisms involve both membrane damage and biological signal interference (Figure 8D). Amphiphilic glycerides mimic membrane lipids and disrupt bilayer organization, increasing permeability. Supported lipid bilayer studies combined with QCM-D measurements show that monoglycerides can cause significant and often irreversible membrane disruption above the critical micelle concentration, accompanied by membrane remodeling (e.g., bud/tubule formation) [85].

In parallel, glycerides interfere with quorum sensing pathways. Consistent with agr-regulated signaling suppression, GML treatment can reduce virulence expression and cooperative behaviors in *S. aureus* [89].

## 6. Synergistic Antibacterial Effects of Composite Biodegradable Films

Single-component biodegradable films often encounter inherent limitations in practical applications. These limitations include restricted antibacterial spectra, difficulty in simultaneously achieving high mechanical strength and barrier performance, and insufficient control over the immobilization and sustained release of active agents. As a result, antibacterial efficacy may decline rapidly due to burst release in the early stage followed by diminished long-term activity.

To overcome these constraints, recent research has increasingly shifted toward composite biodegradable film systems. The central design philosophy of composite films lies in exploiting functional complementarity and interfacial synergy among multiple components, thereby constructing material platforms that combine structural stability with durable antibacterial performance. Such systems have demonstrated enhanced and prolonged inhibition of *S. aureus* compared with single-material counterparts.

### 6.1. Polysaccharide–Protein Composite Films: Microenvironment Regulation and Dual-Barrier Construction

**Figure 9:**
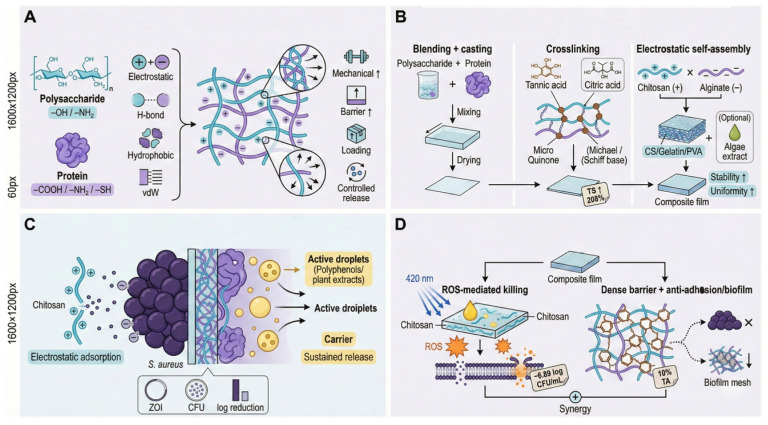
Chemical crosslinking, interfacial complementarity, and synergistic “barrier–killing” mechanisms of polysaccharide–protein composite films. (**A**) Intermolecular interactions; (**B**) film construction strategies; (**C**) synergistic antibacterial performance; (**D**) dual antibacterial pathways.

Polysaccharide–protein composite films are functional biopolymer systems constructed through multiple intermolecular interactions, including electrostatic attraction, hydrogen bonding, hydrophobic interactions, and van der Waals forces. Polysaccharides are typically rich in hydroxyl or cationic amino groups, whereas proteins contain carboxyl, amino, and thiol residues. This complementary functional group distribution enables both simple physical blending and deep molecular integration via chemical crosslinking or electrostatic self-assembly (Figure 9A,B).

Chemical crosslinking is widely employed to reinforce network density and mechanical integrity. For example, soy protein isolate (SPI) crosslinked with dialdehyde starch under tannic acid mediation exhibits a 208% increase in tensile strength compared with pristine protein films. This enhancement is attributed to the oxidation of phenolic hydroxyl groups in tannic acid to quinones, which subsequently undergo Michael addition and Schiff base reactions with protein chains, forming a rigid and densely crosslinked network [90].

Electrostatic self-assembly provides another efficient route for constructing composite films. Positively charged chitosan readily forms strong electrostatic complexes with negatively charged gelatin or sodium alginate. In chitosan/gelatin/poly(vinyl alcohol) ternary systems, electrostatic interactions improve compatibility, regulate pore size, and modulate permeability, creating a stable microenvironment for loading bioactive components such as *Duchesnea indica* extract and improving antimicrobial performance in food applications [91].

Against *S. aureus*, polysaccharide–protein composite films typically exhibit synergistic antibacterial behavior arising from the coupling of direct bactericidal activity and structural barrier effects (Figure 9C,D). On one hand, intrinsic antibacterial properties of polysaccharides (e.g., chitosan) are preserved or amplified, while the composite matrix serves as a reaction platform for external agents such as photosensitizers. For instance, chitosan/curcumin composite films generate abundant reactive oxygen species (ROS) under 420 nm blue light irradiation, leading to oxidative damage to bacterial DNA and proteins and resulting in a 6.89 log CFU/mL reduction in *S. aureus* populations [92].

On the other hand, structural densification induced by crosslinking significantly suppresses bacterial adhesion and migration. In SPI/dialdehyde starch systems, tannic-acid-mediated networks effectively inhibit surface colonization and biofilm formation, with composite films containing 10% tannic acid showing pronounced antibiofilm activity. The integration of “barrier exclusion” and “contact killing” mechanisms renders polysaccharide–protein composites markedly superior to single-component films under complex environmental conditions.

### 6.2. Polysaccharide–Lipid Composite Films: Hydrophilic–Hydrophobic Synergy and Active Barrier Formation

The primary objective of polysaccharide–lipid composite films is to integrate hydrophilic polysaccharide networks with hydrophobic lipid phases, thereby compensating for the inherently poor water vapor barrier properties of polysaccharide films [93]. While some polysaccharides (e.g., chitosan) exhibit baseline antibacterial activity against *S. aureus*, their high polarity leads to moisture absorption and swelling under humid conditions, limiting long-term stability. Incorporation of lipid components—often in the form of essential-oil nanoemulsions—creates discrete hydrophobic domains or interfacial layers that reduce water vapor permeability and enhance structural compactness [94].

**Figure 10:**
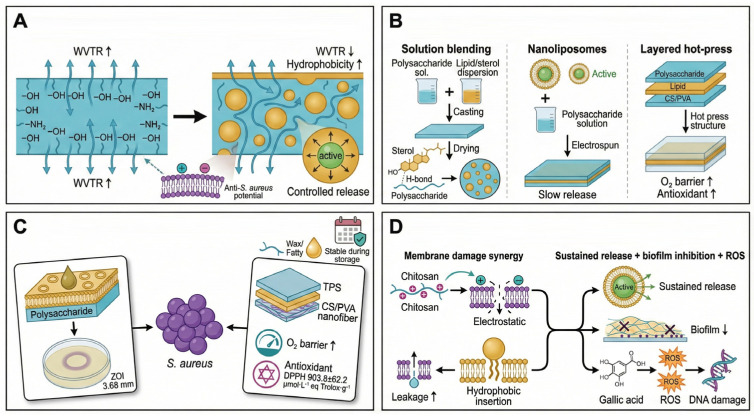
Multiscale interfacial construction, hydrophilic–hydrophobic synergy, and sustained-release mechanisms of polysaccharide–lipid composite films. (**A**) Structural complementarity; (**B**) fabrication strategies; (**C**) antioxidant and long-term antibacterial effects; (**D**) synergistic antibacterial pathways.

Depending on the spatial distribution of lipid phases, three major construction strategies have been developed (Figure 10A,B). Interfacial structuring strategies enable molecular- or microscale dispersion through hydrogen bonding and hydrophobic interactions; for example, bilayer polysaccharide-based films combining extrusion and electrospinning have been developed to tailor interfacial structure and surface uniformity for active packaging [95].

Nanostructured lipid encapsulation represents an advanced strategy. Encapsulation of cumin extract within nanoliposomes, followed by incorporation into nanochitosan-based coatings, enhances protection and sustained release of active agents in food systems [96]. Alternatively, introducing hydrophobic modifiers such as polydimethylsiloxane (PDMS) into polysaccharide-based films can enhance water resistance and barrier performance by creating hydrophobic domains within the matrix [97].

Synergistic antibacterial effects against *S. aureus* arise from coordinated hydrophilic–hydrophobic membrane disruption and lipid-phase-controlled release (Figure 10C,D). Chitosan (via its) cationic groups disrupt bacterial surface charge balance, while lipid domains or hydrophobic antimicrobials insert into lipid bilayers, intensifying membrane disorder. In nanoliposome-chitosan systems, such hydrophilic–hydrophobic synergy can enhance antimicrobial performance by coupling membrane disruption with controlled release [96].

When oxidative components are incorporated (e.g., gallic-acid-grafted chitosan), composite films induce excessive ROS generation, resulting in lipid peroxidation and DNA damage. Microscopy observations confirm severe envelope disruption and intracellular leakage under such synergistic attack [98]. Moreover, lipid microdomains can serve as reservoirs for hydrophobic antimicrobials, supporting sustained activity. Polysaccharide–lipid composites containing beeswax or fatty acids can act as reservoirs for hydrophobic antimicrobials, helping maintain antimicrobial performance during storage by retarding volatilization and diffusion [94,96].

### 6.3. Protein–Lipid Composite Films: Microenvironment Remodeling and Metabolic Targeting

Protein–lipid composite films are designed to integrate hydrophilic protein networks with hydrophobic phases (e.g., lipid domains or hydrophobic biopolyester layers), thereby overcoming the moisture sensitivity and limited vapor barrier performance of protein-based films [99]. While proteins provide excellent film-forming ability and biocompatibility, their polarity promotes swelling and uncontrolled release under high humidity. Lipid incorporation significantly reduces water vapor permeability and establishes hydrophobic microenvironments that stabilize active agents and prolong antibacterial activity [100].

Composite fabrication typically involves multiscale interfacial engineering, including dispersion, assembly, and layered integration. Peptide SA6 was combined with SPI to form oil–water interfacial particles (mean size ~417.4 nm) stabilized mainly by hydrogen bonding; increasing SA6 content reduced polydispersity and creaming index during storage while enhancing antibacterial inhibition [101].

Langmuir–Blodgett (LB) assembly enables precise control of interfacial ordering. MnO_2_ nanowire-templated lipid monolayers transferred onto PDMS substrates generate ordered nanostructures (~80 nm) capable of efficient *S. aureus* capture after antibody functionalization [102]. Emulsion–solvent evaporation and coating strategies further extend industrial applicability. Protein–lipid blending or bilayer structuring has been widely used to improve water resistance and barrier performance of protein-based films, including collagen/gelatin-derived matrices [103].

In coating applications, composite systems enable controlled release kinetics. For instance, antimicrobial peptide OP-145 incorporated into poly(lactic acid)/lecithin coatings on polyurethane surfaces exhibits near zero-order release behavior, with 55% release within 24 h followed by sustained release over seven days, effectively suppressing biofilm formation [104].

**Figure 11:**
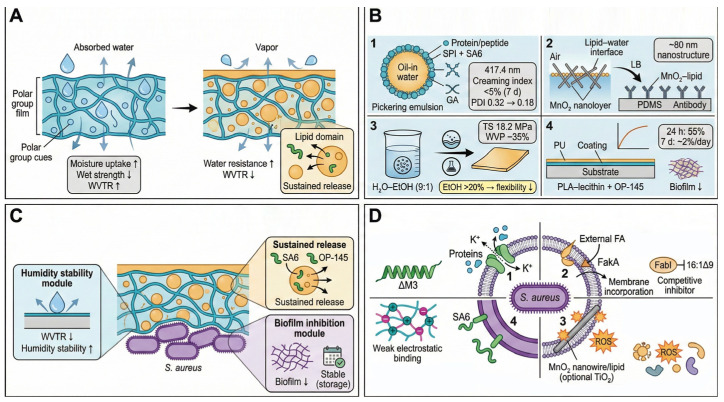
Microenvironment remodeling, fabrication strategies, and multidimensional antibacterial mechanisms of protein–lipid composite films. (**A**) Structural complementarity; (**B**) coating and film formation; (**C**) targeted antibacterial effects; (**D**) multilevel antibacterial pathways.

Antibacterial mechanisms against *S. aureus* involve tight synergy between physical membrane disruption and metabolic interference (Figure 11D). Antimicrobial peptides or unsaturated fatty acids insert into bacterial membranes, forming transmembrane pores. Synthetic peptide ΔM3 exhibits antibacterial activity against *S. aureus* in the micromolar range and induces membrane depolarization and calcein leakage in membrane models, supporting pore-forming membrane disruption [105].

Lipid metabolic interference represents a distinctive mechanism. *S. aureus* relies on fatty acid kinase (FakA) for exogenous fatty acid utilization. Specific lipid components in composite films may act as ineffective substrates, perturbing FakA-mediated exogenous fatty acid utilization and remodeling membrane lipid composition, ultimately restricting bacterial growth [106]. In nanocomposite systems, ROS generation further exacerbates oxidative stress, leading to metabolic collapse [102].

Cell wall–membrane cooperation also contributes to antibacterial efficacy. Protoplast studies reveal reduced sensitivity of wall-deficient cells to peptide SA6, indicating that peptidoglycan structures facilitate initial binding and insertion. Protein matrices additionally suppress bacterial adhesion via electrostatic interactions, amplifying inhibitory effects [103].

### 6.4. Specific Antibacterial Mechanisms of Functional Additives and Nanomaterials

To overcome the performance ceilings of pristine biopolymers, functional additives and nanomaterials enable precise tuning of antibacterial efficacy against *S. aureus* through distinct physicochemical pathways.

(1) Edge-/contact-mediated membrane damage (“nano-knife” and wrapping effects): Graphene and its derivatives can induce physical membrane stress via sharp sheet edges, local contact pressure, and cell wrapping, leading to membrane permeabilization and leakage. Such effects are frequently reported in graphene-containing biopolymer films and composites [51,64,107].

(2) Oxidative stress and ion release: Metal/metal oxide nanomaterials (e.g., ZnO, TiO_2_, iron-oxide-based nanomaterials) inhibit bacteria by generating ROS and/or releasing active ions that disrupt proteins, lipids, and genetic material, thereby collapsing essential metabolic processes [36,56,68,98].

(3) Photo-/catalysis-assisted ROS amplification: Photosensitizers or catalytic nanomaterials incorporated into polymer matrices can amplify ROS under external stimuli (e.g., blue/UVA light), intensifying oxidative damage and enhancing inactivation efficiency [38,92].

(4) Carrier-enabled delivery of small-molecule antimicrobials: Encapsulation and controlled migration of essential oils, organic acids, and other bioactives within polymer–nanomaterial microenvironments can improve stability and prolong antibacterial duration by regulating partitioning and diffusion [61,63,72,94].

### 6.5. Enhancement of Barrier Properties Through Nanomaterial Integration

Beyond antibacterial activity, nanomaterial integration is a key strategy to overcome the inherent gas/moisture sensitivity of biopolymer films:

(1) Tortuous-path mechanism: Platelet-like or high-aspect-ratio fillers (e.g., nanocellulose, graphene-derivatives, nanostructured oxides) create a staggered diffusion pathway that retards O_2_/H_2_O transport, reducing permeability and stabilizing film performance [36,40,107].

(2) Interfacial sealing and network densification: Nanofillers and crosslinking can reduce free volume and strengthen interfacial interactions, producing denser micro-networks with improved barrier integrity [36,90,97].

(3) Hydrophobicity tuning: Hydrophobic nano-additives and wax/lipid-functionalized systems can increase surface contact angle and reduce moisture uptake, which helps maintain structural stability under humid conditions [82,97].

## 7. Core Principles and Regulatory Mechanisms Governing Antibacterial Performance

Comprehensive analysis of existing studies indicates that the antibacterial performance of biodegradable films against *S. aureus* is not governed by isolated material parameters, but rather follows generalizable material–biological interaction principles. Specifically, the molecular structure of the film matrix defines a baseline antibacterial potential, composite or modification strategies introduce synergistic enhancement through mechanism complementarity, and environmental factors dynamically regulate antibacterial durability by modulating material responsiveness and active agent migration.

These phenomena can be fundamentally interpreted as a process of optimizing compatibility between material molecular features and antibacterial mechanisms [107]. Only when active sites, network architecture, and action pathways are well matched can antibacterial strength, persistence, and application feasibility be simultaneously achieved.

To resolve the apparent inconsistency between the global three-pathway framework and the material-specific mechanisms discussed throughout the manuscript, we provide a hierarchical mapping (Table 3) that links global categories to sub-mechanisms and the experimental endpoints used to validate them.

### 7.1. Molecular Structure of the Matrix Determines the Baseline Antibacterial Potential

The intrinsic antibacterial potential of biodegradable films is primarily determined by the molecular structure of the matrix material. Key structural parameters include the type and density of functional groups, surface charge characteristics, hydrophobicity, and the compactness of the film-forming network. Collectively, these parameters establish a baseline level of antibacterial activity that constrains subsequent performance enhancement.

For polysaccharide-based matrices, cationic group density is a decisive factor. Chitosan serves as a representative example: when the degree of deacetylation (DD) exceeds 85%, the high density of protonated amino groups enables strong electrostatic disruption of the anionic cell wall of *S. aureus*, often achieving high inhibition (frequently >90% under appropriate conditions) [13]. In contrast, neutral polysaccharides such as cellulose and starch lack membrane-active charged groups, and they typically exhibit little to no intrinsic antibacterial activity. As a result, these materials rely heavily on external functionalization [109], such as nanoparticle loading or essential oil incorporation, to compensate for their limited activity. This relationship has been summarized as a positive correlation between cationic group density and antibacterial potential [109].

Protein-based matrices exhibit antibacterial behavior governed by the interplay between functional residues and three-dimensional network organization. Amino acid side chains provide interaction sites, whereas network architecture determines their effective presentation and persistence. For example, collagen films utilize porous structures to host ROS-generating nanoparticles [51]; whey protein films exploit protein network structure to modulate the migration/release of incorporated antimicrobials (e.g., organic acids) and extend antibacterial duration [61]; whey-protein films incorporating other antimicrobial additives (e.g., liquid smoke) further illustrate how protein–additive interactions balance activity and durability [62]; and soy protein globular structures encapsulate essential oils through hydrophobic interactions to support encapsulation and controlled release [110]. Consequently, the advantage of protein-based systems lies not in strong intrinsic antibacterial activity, but in their exceptional structural designability and delivery controllability.

For lipid-based matrices, antibacterial activity depends on a balance between fatty acid chain length and hydrophobicity. Medium-chain fatty acids, exemplified by lauric acid (C12), achieve optimal membrane insertion capability, exhibiting MIC values as low as 156 µg/mL against *S. aureus* [74]. In contrast, fatty acids with different chain structures can exhibit markedly different membrane interactions; for example, palmitoleic acid (C16:1Δ9) rapidly induces membrane depolarization and leakage of intracellular solutes in *S. aureus* [75]. Unsaturation further enhances membrane perturbation; for instance, myristoleic acid inhibits biofilm formation at concentrations as low as 1 µg/mL [76]. This indicates that the effectiveness of lipid-based systems does not simply increase with greater hydrophobicity; rather, there exists a structural window that can be deliberately exploited through material design. The importance of structure–property matching has been demonstrated in other fields as well. Analogously, particle-size fractionation in heavy-metal-contaminated soil remediation can improve process efficiency by precisely controlling key structural parameters, reinforcing the general design rule that performance can be optimized via targeted structural tuning [111].

### 7.2. Complementary Synergy Between Antibacterial Agents and Matrix Materials

When single matrices exhibit inherent structural limitations, composite systems frequently achieve performance breakthroughs through mechanism complementarity rather than simple additive effects. The effectiveness of synergy depends critically on the degree of matching between matrix functions and antibacterial agent action pathways.

Charge complementarity represents one of the most common sources of synergy. For example, electrostatic interactions between cationic chitosan and protein-based components such as gelatin (often with poly(vinyl alcohol) as a compatibilizer) can improve film integrity and compatibility, creating a stable microenvironment for incorporating bioactive plant extracts (e.g., *Duchesnea indica* extract) and enhancing antimicrobial performance in food applications [91]. Similarly, quaternized soy protein introduces stable positive charges that strengthen electrostatic interactions with bacterial surfaces, enabling inhibition rates up to ~98% under appropriate conditions [71].

Mechanistic complementarity further manifests as the integration of physical barrier effects with active antibacterial mechanisms. In polysaccharide–lipid composite films, cationic polysaccharides provide contact-active antibacterial effects, while lipid domains create hydrophobic barriers that reduce bacterial adhesion. Superhydrophobic beeswax-based surfaces (contact angle > 150°) significantly reduce bacterial attachment, while residual chitosan amino groups disrupt adherent cells [82]. In protein–nanomaterial composites, protein matrices can act as carrier platforms for antibacterial nanofillers (e.g., graphene and iron oxide nanoparticles), thereby enhancing antibacterial activity against *S. aureus* [51].

Controlled-release regulation is central to achieving sustained antibacterial performance. In chitosan-based systems, incorporating clove essential oil via microemulsion loading can improve retention and sustained antimicrobial efficacy, supporting extended preservation performance in pork products [108]. Whey protein network structures can modulate the migration/release of incorporated organic acids, contributing to prolonged antimicrobial efficacy and improved shelf-life performance in food systems [61]. These findings demonstrate that when matrix architectures enable “retention–release” control without compromising interfacial interactions, antibacterial durability can be systematically engineered.

### 7.3. Dynamic Regulation of Antibacterial Performance by Environmental Factors

The antibacterial efficacy of biodegradable films is inherently dynamic and sensitive to environmental conditions. Factors such as pH and temperature modulate the ionization state of functional groups, network compactness, and diffusion pathways of active agents, thereby regulating antibacterial performance over time.

pH primarily influences antibacterial activity by altering matrix ionization. In chitosan films, acidic conditions (typically below ~pH 6) promote amino group protonation, generally strengthening electrostatic interactions with *S. aureus* and enhancing antibacterial activity; under near-neutral conditions, deprotonation can weaken this effect [13]. In contrast, carboxymethyl chitosan can retain better water solubility and antibacterial effectiveness around neutral pH due to the presence of carboxyl groups [13]. These observations confirm that pH regulates antibacterial performance by controlling the ionization state of active functional groups [109].

Temperature influences antibacterial behavior through structural evolution and diffusion kinetics. Processing temperature during film preparation can alter protein denaturation and network formation, thereby affecting film properties and the migration of incorporated antimicrobials [62]. Similarly, the thermal history of starch-based films (e.g., gelatinization and subsequent structural reorganization) governs crystallinity; increased crystallinity can delay antimicrobial release and prolong antibacterial activity [112].

These findings highlight the necessity of optimizing processing, storage, and application temperature windows in conjunction with matrix structural stability. Antibacterial performance should therefore be evaluated as a function of environmental responsiveness rather than as a static material property.

### 7.4. Food-Safety Relevance of Enterotoxins and Practical Control Thresholds

A key food-safety feature distinguishing *Staphylococcus aureus* from many other foodborne hazards is its ability to produce heat-stable staphylococcal enterotoxins (SEs). Once preformed in food, SEs may remain biologically active even after thermal processing; therefore, inhibiting bacterial growth does not necessarily guarantee toxin risk mitigation if toxin has already accumulated. From a packaging perspective, the primary preventive value of antimicrobial films lies in maintaining *S. aureus* populations below toxin-production–associated thresholds during storage and distribution, rather than attempting to “remove” toxins after formation.

In practice, toxin risk assessment requires coupling microbial control with appropriate detection and verification. Common approaches include immunoassays (e.g., ELISA or lateral-flow tests) for SE screening, targeted mass spectrometry (LC–MS/MS) for confirmatory profiling, and nucleic-acid-based assays that support strain characterization (noting that gene presence does not equal toxin production). Integrating such detection workflows with antimicrobial packaging trials (especially in real food matrices) enables a clearer linkage between structure–mechanism design and food-safety endpoints.

These considerations also shape design priorities for next-generation active packaging. Films should emphasize (i) early-stage suppression and antibiofilm activity to prevent the population from reaching toxin-risk conditions, (ii) moisture/oxygen barrier stability to avoid environmental drift that promotes growth, and (iii) controlled-release kinetics that sustain inhibition throughout the intended shelf life. Where feasible, multifunctional designs that combine growth suppression with toxin-focused monitoring (or adsorption/neutralization strategies) would further strengthen real-world food-safety relevance.

## 8. Conclusions and Perspectives

### Final Conclusions

The systematic evolution of biodegradable films from passive physical barriers to active antimicrobial platforms is fundamentally driven by the synergistic optimization of material structures and biological action pathways. This review clarifies that the baseline antibacterial potential against *S. aureus* is primarily governed by the molecular features of the polymer matrix, such as the cationic charge density in polysaccharides or the chain-length- and unsaturation-dependent hydrophobicity in lipid systems. By integrating these intrinsic properties with composite strategies—including charge complementarity, amphiphilic assembly, and controlled-release kinetics—hybrid materials successfully overcome the performance ceilings of single-component matrices. Furthermore, the dynamic nature of these systems ensures that their inhibitory efficacy is actively modulated by environmental fluctuations in pH and temperature, which regulate both matrix ionization and active-agent migration.

Looking forward, the transition of these high-performance films from laboratory prototypes to industrial reality requires addressing several critical engineering bottlenecks. A primary challenge lies in resolving the inherent trade-off between antimicrobial loading and mechanical integrity, where hierarchical structural engineering and crosslinking offer promising routes to reinforce load-bearing frameworks without compromising bioactivity. Moreover, the development of intelligent, stimuli-responsive systems that trigger release in direct response to the local biochemical cues associated with *S. aureus* growth—such as localized acidification or enzymatic secretion—represents the next frontier in maximizing bioavailability. Finally, future efforts must prioritize the development of green, solvent-free manufacturing routes, such as extrusion or blow molding, supported by rigorous safety assessments and migration models. Together, these advancements will establish a robust theoretical and practical foundation for the next generation of active packaging to safeguard global food safety.

## Figures and Tables

**Table 1:** Key characteristics of antibacterial films based on several cellulose materials.

Material Type	Key Characteristics	Antibacterial Mode of Action	Application Advantages and Challenges	Refs.
Cellulose derivatives (e.g., CMC)	High water solubility, smooth film formation; good affinity for polar compounds.	Primarily used as a carrier matrix for antibacterial agents (e.g., essential oils and silver nanoparticles).	Good film-forming ability but poor water resistance and limited mechanical strength requiring further improvement.	[33,34,39]
Bacterial cellulose (BC)	High purity; high water-holding capacity; 3D nanofibrillar network.	Excellent physical scaffold for immobilizing active agents; composite formulations can provide synergistic antibacterial effects.	Excellent biocompatibility but relatively high production costs limits large-scale application.	[37,38]
Nanofibrillated cellulose (NFC)	High aspect ratio; forms a dense hydrogen-bonded network.	Forms a compact structure that limits mass transfer and permeation; high surface area supports loading/anchoring of antimicrobial components.	Enhances mechanical and barrier performance, but dispersion stability remains a key challenge.	[40]

**Table 2:** Comparative analysis of protein-based antibacterial films.

	Core Mechanism	Key Advantages	Major Challenges	References
Collagen	Nanoparticle carrier; ROS generation & electrostatic adsorption	High biocompatibility; Ideal for biomedical scaffolds (e.g., wound dressings)	High cost; Religious/dietary restrictions; Nanotoxicity risks	[47,48,51,56,57]
Whey Protein	Controlled release of organic acids & Hydrophobic EO encapsulation	Excellent food-grade coating; Extends shelf life of dairy/meat products	Poor moisture resistance; Mechanical fragility in high humidity	[58,59,60,61,62,63]
Soy Protein	Endogenous peptides (GBP); Inorganic synergism (ZnO) & Quaternization	Low cost; Highly sustainable for eco-friendly food packaging	Beany off-flavors; Trade-off between modification and biodegradability	[65,68,70,71,72]

**Table 3:** Hierarchical mapping of antibacterial mechanisms against *S. aureus*.

Global Category (3-Pathway Framework)	Material-Specific sub-Mechanisms	Typical Material/Structure Examples	Primary Experimental Endpoints (Recommended)	Key Test-Condition Fields to Report (for Comparability)	References
Physical barrier effects	Reduced water uptake/swelling; reduced mass transfer (H_2_O/O_2_); tortuous diffusion path	Lipid/wax domains; PDMS-modified polysaccharide films; nanocellulose/nanofillers densifying networks	WVP/OTR; moisture sorption; contact angle; log CFU reduction in food matrix	Film thickness; humidity/temperature; food matrix type; storage time	[82,94,97]
	Anti-adhesive/superhydrophobic interface	Beeswax–SiO_2_ superhydrophobic coatings	Adhesion assays (CFU on surface); contact angle; biofilm biomass	Surface roughness; contact angle; inoculum; exposure time	[82]
Chemical interference	Electrostatic disruption of cell envelope	High-DD chitosan; quaternized proteins	MIC/MBC; membrane depolarization; ATP/K^+^ leakage	pH; ionic strength; DD/charge density; strain; assay method	[13,71]
	Membrane insertion/disordering by amphiphiles	Fatty acids (e.g., C16:1Δ9); monoglycerides (GML)	Membrane depolarization; ATP leakage; time-kill; lipid-bilayer model assays	Fatty acid species; concentration; CMC; exposure time; temperature	[75,85,105]
	ROS-mediated oxidative damage (with/without light)	ZnO/TiO_2_; photodynamic films; chitosan–gallic acid (UVA)	ROS assays; log CFU reduction; microscopy evidence; oxidative damage markers	Light wavelength/intensity/time (if used); oxygen availability; loading	[68,92,98]
	Ion release and intracellular interference	ZnO releasing Zn^2+^; metal oxide systems	MIC/MBC; DNA/protein damage markers; time-kill	Ion concentration; medium composition; pH	[68]
	Controlled release / diffusion regulation	WPI films with organic acids; nanoemulsion reservoirs; microemulsion-loaded systems	Release kinetics; log CFU reduction in food matrix; time-kill	Thickness; loading; release medium; temperature/humidity	[61,94,108]
Biological regulation	QS inhibition and virulence downregulation	Unsaturated fatty acids (petroselinic acid); GML/lauric acid signaling inhibition	RT-qPCR (agrA/hla/nuc); toxin/virulence assays; biofilm biomass	Strain; growth phase; gene targets; normalization method	[77,89]
	Biofilm matrix destabilization (EPS disruption)	Antibacterial fatty acids dispersing established biofilms	Biofilm biomass (CV); CLSM; viable counts in	Biofilm model; surface; incubation time; quantification method	[73,77]

Note: ZOI values are diffusion-dependent and should not be ranked across studies without standardized conditions; therefore, MIC/MBC, log CFU reduction, time-kill assays, and food-matrix tests are prioritized whenever available.

## Data Availability

No new data were created or analyzed in this study. Data sharing is not applicable to this article.
